# Mental Models of Attachment in Adoptive Parents and Children: The Case of Institutionalized and Adopted Young Adults

**DOI:** 10.3390/ijerph22050776

**Published:** 2025-05-14

**Authors:** Angelica Arace, Protima Agostini, Laura Elvira Prino

**Affiliations:** Department of Philosophy and Education Sciences, University of Turin, 10124 Turin, Italy; protima.agostini@unito.it (P.A.); lauraelvira.prino@unito.it (L.E.P.)

**Keywords:** adoption, attachment, institutionalization, young adult, adoptive parents, late-adopted, AAI

## Abstract

The international adoption of early institutionalized children offers the opportunity to examine the quality of mental representations of attachment and their possible revision post-adoption, thus contributing to the debate on the continuity/discontinuity of internal working models and the intergenerational transmission of attachment. The main aim of this study was to investigate how early institutionalization affects the IWMs of adopted children and whether there was a relation between the IWMs of adoptive parents and those of their children. Participating in the study were 39 young adults (male: 15; female: 24) and their adoptive parents (N = 72): adoptees’ IWMs were assessed with the SAT, while parents were administered the AAI. The percentage of insecure and especially disorganized attachments in adoptees differs significantly from the normative data of the reference population. The IWMs of adoptive parents only partially reflect the normative distribution of the non-clinical adult population, with dismissing models being overrepresented. There is no consistency between the IWMs of adoptees and those of adoptive parents. This study highlights the negative effects, even in the long term, of early experiences of emotional deprivation and the stabilization of insecure attachment patterns in the absence of caregivers who can act as a secure base that enables children to come to terms with their traumatic past.

## 1. Introduction

### 1.1. Adoption: Recovery Process After Early Adversity

Loss of parental care and early institutionalization are central issues in adoption, particularly in international and late adoptions [1]. Indeed, children in these situations have often experienced numerous adversities and traumas in the early years of their lives, including separation from their biological parents, multiple changes in primary caregivers, and physical and psychological neglect. Due to these adversities, they are exposed to greater mental health risk factors and exhibit more adjustment difficulties than their non-adopted peers [2,3,4].

Among adopted children, those who have experienced prenatal and postnatal adversity and trauma prior to adoption have a higher risk of maladjustment and adoption failure [5,6]. Especially in recent years, the literature has increasingly emphasized the issues related to trauma, loss, attachment, identity, and intimacy that can accompany the adoption process [7,8], and adoption is no longer described solely as a win–win situation [9] in which the needs of adoptive children, adoptive parents, and biological parents are met. The relationship between adoption and trauma primarily reflects a framework of adversity and trauma that occurs for these children prior to entering adoption, while adoption itself allows for a degree of recovery over time [10].

Adoption is therefore an intervention that can interrupt a painful and maladaptive growth path and promote the well-being of children with a rehabilitative function of their developmental opportunities [11]. Indeed, the literature consistently emphasizes the greater physical, cognitive, and psychological well-being and development of adopted children compared to children who remain in disadvantaged situations [2,5,12].

In order to promote recovery from trauma, it is crucial that children can have new positive relationship experiences with their adoptive parents [12,13,14]. This requires creating a stable environment with adults who are able to understand the complexity of their children and provide them with the kind of care that fosters secure attachment, good self-representation, and a strong sense of belonging [3,15,16,17,18,19]. A relationship with adoptive parents or other caregivers that is based on sensitive responsiveness, stability, security, and family membership can help children to revise their insecure internal attachment working models, thus interrupting the intergenerational transmission of deprivation and redirecting their developmental trajectories, as also shown by studies of children with a background of abuse, neglect, and psychosocial adversity who have grown up in foster care. [20].

### 1.2. Attachment Theory and the Adoption Process

Attachment theory provides a valid and reliable framework for understanding the impact of close relationships on typical and atypical trajectories of social and emotional development across the lifespan and for examining relationships in adoptive families by analyzing the effects of separation, loss of biological parents, and possible early institutionalization on the formation of self-representation [21,22]. While most children experience continuity in their attachment relationships throughout childhood, the development of adopted children is characterized by a radical discontinuity in primary care and relationships. This discontinuity is even more pronounced in international adoptions, as many children spend a relatively long time in residential care before being adopted [23].

According to Bowlby [21], children build internal working models (IWMs), i.e., representations of self and other in relationships, based on early social interactions with their caregivers. Attachment research in the context of adoption contributes to the debate about one of the central assumptions of attachment theory, namely whether and to what extent internal working models persist throughout childhood and adolescence into adulthood [24,25] or whether they are malleable and can be revised on the basis of later interpersonal experiences [16,26]. While there is a broad consensus that individuals’ attachment representations respond adaptively to changes in the caregiving environment [27], there is also evidence that experiences in early attachment relationships leave a lasting impression that shapes later development even in the face of changes in the caregiving context [28].

In adopted children, internal working models within a short time after adoption are usually characterized by a devalued and unlovable self-image and an image of the other as dismissive and sometimes threatening [1,16]. Later internationally adopted children also lived in institutions and often experienced additional trauma and did not have the opportunity to form meaningful attachment relationships due to limited contact with their caregivers [29,30,31,32]. Adopted children, especially late-placed ones, are thought to be at higher risk of developing insecure or disorganized attachments due to adverse experiences such as abandonment, loss, neglect, abuse, and maltreatment in their family of origin or in institutions [33]. However, as mentioned earlier, positive experiences in a responsive caregiving context with adoptive parents may enable them to shift their insecure or disorganized IWMs toward security [34,35].

Adoption can thus be described as a ’natural experiment‘ [12] in which IWMs can change due to the experience of positive attachment relationships that can compensate for the trauma experienced in early interactions. Indeed, a positive change in attachment toward security has been observed in adopted children compared to peers in foster care and residential care [24,30,36,37,38,39].

However, research analyzing and comparing attachment between adopted and non-adopted children comes to contradictory conclusions. Many studies report a higher frequency of insecure and disorganized attachment relationships in adopted children [40,41], which is consistent with the hypothesis that early experiences of loss and separation can influence the quality of later attachment relationships [38]. Adopted individuals with an unfavorable history are more likely to develop insecure representations than non-adopted individuals, as assessed with story–stem methods [42,43,44,45], autobiographical narratives [46,47], and family drawings [48,49].

On the other hand, some studies find that attachment security does not differ between adopted and non-adopted children [34,35,50], emphasizing how positive experiences with adoptive parents can promote a discontinuity in internal working models and attachment relationships toward security. This discontinuity is most evident between childhood and adolescence, as the attachment system changes and reorganizes during this period of transition to adulthood [51,52].

In the debate about continuity and discontinuity of attachment behavior and internal working models in adoptive families, the developmental trajectories of late-adopted children [53] play an important role. Once they become adolescents or young adults, they integrate internal working models that are not always coherent given early experiences of institutionalization and relationship disruption with at least one caregiver, factors that may increase their psychoaffective vulnerability [7,16]. From this perspective, it is useful to consider the behavioral and representational levels of attachment separately, as discrepancies can be found. When only behavior is assessed, late-adopted children often show some signs of security, although it may take longer for their internal working models to change [7] and the shift toward more positive representations of self and others may persist into adulthood [16].

### 1.3. Risk and Protective Factors

There are several risk and protective factors that can influence the adoption process and may explain the contradictory results described above. The risk factors most commonly considered in research are older age at the time of adoption, older age at the time of separation from the biological family, length of previous institutionalization, and any deprivation and trauma in the time leading up to adoption [54]. On the other hand, an autonomous state of mind in adoptive parents is an important protective factor, although some individual characteristics such as temperament and intelligence should not be disregarded.

In particular, the age of adoption is one of the most critical factors correlated with psychological problems [2,55] and with greater difficulties in changing attachment behaviors and especially attachment representations [7,53]. In fact, many studies have shown that insecurity and disorganization in attachment behavior or attachment representation are more pronounced in late-adopted children than in early-placed children and in children who grew up with their biological family [12,32,40,43,45,49,56,57,58].

Late adoption is also often associated with other risk factors, such as a prolonged period of institutionalization, trauma, or other adversities in the early years [4,13]. Indeed, research shows lower well-being and adjustment in late-adopted children, which is reflected not only in greater attachment insecurity, but also in greater vulnerability to emotional and behavioral difficulties [59] and lower cognitive and language skills [60].

Among the protective factors that can reduce the impact of risk factors by increasing the resilience of adopted children, empathic and sensitive care and stimulation by alternative parental figures and increased permanence in the adoptive family play an important role [32]. Some research has found that the security of adoptive mothers’ internal working models is an important factor that can influence the possibility of building a secure relationship with the adoptive family [50,61,62]. Longitudinal studies suggested that a large number of adopted children ‘gain security’ some time after adoption, particularly when placed with adoptive parents who provided a high quality of care that was associated with a sense of security in relation to their own attachment experiences [18,41,58,63,64]. With time after adoption, for example, symptoms of attachment disorders decreased in late-adopted children [18,57] and parents were more frequently portrayed as helpful and responsive [56]. However, these changes exclusively characterized children adopted by parents who had demonstrated autonomous and secure mental states in response to the Adult Attachment Interview (AAI) at the time of adoption [35]. In other words, children may adopt new positive representations of current experiences, but older negative experiences may persist unless there is a ‘secure’ adoptive parent in the children’s lives who gently helps them to let go of their thoughts and feelings about their negative past.

These findings are related to two other important questions, namely whether the mental representations of attachment in adoptive parents differ from those of non-adoptive parents and whether concordance can be observed between the representations of attachment in adoptive parents and adoptive children in line with what is known in the literature about the process of intergenerational transmission of attachment. Regarding the first question, nearly 75% of parents who adopted internationally and couples who are seeking to adopt domestically are classified as having an autonomous state of mind during the Adult Attachment Interview [38,39]. This estimate is substantially higher than the normative base rate of 55–60% for low-risk mothers [65]. Regarding the second question, research with adoptive families provides a unique opportunity to test the process of intergenerational transmission among genetically unrelated parent–child pairs, a question on which results in the literature are often inconsistent.

Finally, an additional protective factor for the well-being and adjustment of adoptees and their families that recent research emphasizes is effective, open, and sensitive communication about adoption [2,66,67,68], in contrast to common practices for much of the 20th century, which assumed that secrecy and confidentiality were essential features of family communication about adoption [69].

### 1.4. Current Study

Many of the studies examined focus on the few years after placement of adopted children, usually during middle childhood, while few studies examine what happens in the later stages, such as late childhood and adolescence [70], and even fewer studies have been conducted on adulthood. Given this gap in previous research on adolescence and young adulthood, our study aims to fill it, at least partially, and is part of the line of research designed to examine the medium- and long-term effects of adoption. Since the quality of relationships and representations of attachment in adoptive families are important for the well-being and adjustment of adoptees not only in childhood, this research aims to investigate the mental representations of attachment in young adults who were exposed to the loss of family relationships and institutionalization in early childhood and were subsequently adopted by Italian couples.

The main aims of this study are to analyze the internal working models of adopted young adults who have experienced early affective deprivation and compare the results with those of other national and international studies, to analyze the internal working models of adoptive parents and compare the results with those of clinical and non-clinical adult populations, and to investigate the presence or absence of a correlation between the internal working models of children and adoptive parents. As mentioned earlier, several authors [71,72,73,74,75] identify the placement of children who have had troubled histories and severe neglect experiences in adoptive families as one of the strongest factors in the potential reworking and restructuring of attachment patterns that can break the intergenerational transmission of the ’cycle of abuse.’ The literature [76,77,78], given the difficulties and psychological vulnerabilities of children placed for adoption, emphasizes the importance of quality parenting by adoptive parents, both to prevent adoption failure and to provide secure attachment for children who have largely insecure attachments [79,80,81]. Furthermore, our study aims to investigate the protective role of the quantity and quality of communication on the topic of adoption [4,66] and its possible correlation with the representation of attachment in young adult adoptees. Open, sincere, and sensitive communication about the adoption process may facilitate self-reflection, the construction of a more coherent account of self and one’s history, identity by linking past, present, and even future in a coherent and integrated narrative. This process is typical of adolescence and early adulthood [4].

Therefore, the specific aims of our work follow:

- Determine the distribution of attachment patterns in adoptees and to compare the results with normative data from Italian and international adolescents and young adults who grew up in their families of origin;
- Analyze differences between early and late adoptees;
- Determine the attachment style of adoptive parents;
- Analyze the correlation between parents’ and children’s mental representations of attachment;
- Investigate the quality of family communication about adoption and its relationship to children’s attachment representations.

## 2. Materials and Methods

### 2.1. Participants

Thirty-nine young adults (male: N = 15, 38.5%; female: N = 24, 61.5%) participated in the study, having an average age of 22.95 years (sd = 3.84), coming from families with a medium to high socio-cultural level, living in a large northern Italian city, and mostly adopted before the age of 3 years (Table 1). They were all international adoptions: from countries in India and Indonesia (N = 34, 89.5%), South America (N = 3, 7.9%), and Eastern Europe (N = 1, 2.6%). Of 1 participant, the country of origin and age at the time of adoption were unknown.

The composition of the survey sample reflects Italian data on intercountry adoptions, which show that India is still the first country of origin of adopted children, followed by Eastern European countries, such as Hungary, and Latin American countries, such as Colombia [82]. All adopted children came from early experiences of institutionalization, since abandonment has represented over the years and still represents the main motivation behind adoptions from Asian and South American countries [82]. In 38.5% of cases, the participants were only children, in 53.8% of cases they had other adopted siblings, and in 7.7% of cases they had other biological siblings. The percentage relating to the presence of more than one adopted sibling differs from the average Italian data in which the highest percentage of international adoption requests concerns the adoption of a single child [82].

Most of the young people adopted had a high school diploma (70.3%) and many were still studying at the time of the survey (students: 63.2%; employed: 31.6%; unemployed: 5.3%). In 92.3% of cases, they were still living with their parents.

In 69.2% of cases, the adoption was desired and planned by both parents, while in 25.6% of cases it was only desired by the mother. Adoption took place on average 6.31 years after marriage (sd = 3.99), which corresponds to the fact that the class with the highest frequency in Italy continues to be couples married for 3 to 6 years, followed by couples with 7 to 10 years of marriage [82].

As for the parents, at the time of the survey the mothers had an average age of 57.14 (N = 37; sd = 5.98), the fathers an average age of 59.51 (N = 35; sd = 5.93). Most mothers (66.6%) had an average level of education (high school diploma: 33.3%; college degree: 33.3%), as did 79.5 percent of the fathers (high school diploma: 46.2%; college degree: 33.3%). The distribution of educational qualifications confirms an average cultural level among adoptive couples that is decidedly higher than that found in the reference Italian population as a whole; in this regard, the spread of university degrees in the Italian population in the corresponding age bracket affects on average just over 20% of the subjects [82]. Overall, the socio-cultural and economic level of the families in the sample is consistent with Italian data on international adoptions, where, for example, intellectual, scientific, and highly specialized occupations are the most common employment conditions for both husbands and wives [82]. In 15.4% of cases (N = 6), one parent was deceased. In only 5.1% of cases (N = 2) did the parents divorce after the adoption.

### 2.2. Measures

The mental representation of attachment in children was assessed using the semiprojective instrument Separation Anxiety Test—SAT [83] in the version modified by Attili [84] and adapted to the Italian context for the hypothetical child. The instrument consists of twelve vignettes (six for girls and six for boys) depicting scenes of mild separation (for example: *This is the first day of school. Here are the teacher and the classmates. The mother has just dropped the child off.*) and strong separation (for example: *The parents go away for two weeks and leave the child at home. Before they leave, however, they give them a nice present. Here they say goodbye.*) between a school-age child and their parents. The hypothetical child’s version includes four questions about the emotions that the interviewee thinks the child is feeling (*What do you think this child is feeling?*), why the child is feeling these emotions (*Why do you think this child is feeling this way?*), coping strategies the child might use (*What do you think this child is doing now?*), and possible reactions to the reunion with parents (*What do you think this child will do when mom/parents return?*). Each response is scored and the total score is categorized according to the four attachment categories: secure (B), resistant (C), avoidant (A), or at risk of disorganization (D). The Italian version [84] showed satisfactory concordant validity (r = 0.77; *p* < 0.001) with Klagsbrun and Bowlby’s coding system, high inter-rater reliability (Cohen’s k = 0.80; *p* < 0.001) and adequate test—retest correlation (r = 0.75; *p* < 0.001). In this study, two independent and appropriately trained raters coded each response, and high inter-rater agreement (95%) was found. In situations where the two coders disagreed, they compared each other and eventually came to a common conclusion.

The current mental state in relation to parental attachment was assessed using the Adult Attachment Interview [85], a semi-structured interview protocol consisting of 20 questions and lasting approximately one to one and a half hours. The interview focuses on previous experiences in the attachment relationship with parents in childhood. In this study, the interviews were recorded, transcribed verbatim, and analyzed separately by two experts. The interviews were coded according to the method of Main and Goldwyn [86], which allows the assignment of the subject to one of the 4 categories identified by the authors: F (Secure-Free/Autonomous), Ds (Dismissing), E (Entangled–Preoccupied), and U (Unresolved/Disorganized). The inter-rater reliability was high (Cohen’s k = 0.79). In the event of discrepancies, they compared each other and finally came to a common conclusion. The AAI is one of the most widely used measures for studying attachment representations in adulthood. In an Italian meta-analysis, the AAI has shown test–retest stability from 70% (three-ways) to 95% (secure–insecure) over a period of 4 years [87].

The Italian versions of the SAT and the AAI are frequently used in both research and clinical samples to assess attachment. SAT was chosen because the issue of separation and abandonment by parents is a central theme in the history of adopted children. The AAI, on the other hand, was considered too invasive in view of the children’s traumatic history and was only used for the parents. For the same reason, the hypothetical child version of the SAT was also used.

In addition, two ad hoc questionnaires with closed-or-open questions were used for this study. The first, administered to parents, mainly aims to explore memories and feelings related to the transition to adoptive parenthood. For example, parents were asked to write down their first four memories of meeting their adopted child, their first four memories of going home with the child, and the first days of living together, examples of episodes of discomfort that characterized their child’s growing up, and the emotions triggered by their child’s request to reconstruct their pre-adoption and adoption history.

The second questionnaire given to the children aimed to explore memories, emotions, and attitudes toward the adoption journey, the adoptive family, and the family of origin. Specifically, the adopted children were asked how often the topic of adoption came up in conversations with their adoptive parents, whether they remembered some moments of communication about their adoption and the emotions it triggered, whether they had a desire to know their origins, and whether they had difficulty telling other people outside the family (e.g., friends or teachers) about their adoptive status.

### 2.3. Procedures

The research protocol was submitted to the University Ethics Committee for evaluation. After their approval, adoptive families were recruited by sending them a letter of invitation to participate in the study through authorized agencies that assist parents in international adoption procedures. Inclusion criteria were a family with experience of international adoption and with adopted children who were at least 18 years old at the time of the study. No exclusion criteria were applied. Of 40 families contacted, 31 agreed to participate in the study and only families who gave their informed consent were contacted.

Both the SAT and the AAI were administered at the participants’ homes in a separate room to ensure maximum privacy, while the questionnaires were filled out by the participants individually. The administration and coding of the instruments was carried out by trained, blinded researchers. The audio recordings and questionnaires were collected without personal identification data. Only alphanumeric codes were used to match the parents’ and children’s protocols.

### 2.4. Data Analysis

The data analysis was performed with the statistical program IBM SPSS Statistics version 29. Data were analyzed using non-parametric statistical tests appropriate for simple size and type of dependent and independent variables. Exact statistical tests were used for categorical and/or dichotomous variables, which allow working with a small number of observations. Specifically, the following tests were used: Fisher’s exact test for variables in independent samples when the expected frequency of at least one cell was less than 5; the exact chi-square for categorical variables (non-dichotomous) in independent samples. The significance level was *p* < 0.05 for all analyses. The mental representations of attachment in adoptees were examined and associations with gender and age at adoption were analyzed. In analyses that considered age at adoption, the only missing case was excluded. The mental representations of attachment in adoptive mothers and fathers and their degree of concordance with their children’s internal working models were also investigated. In accordance with other studies [11,50], the analyses were performed using both the four-way classification of the attachment system and the two-way classification (secure vs. insecure). This is because the two-way classification allows differences between sub-samples to be identified with greater statistical significance in small-sample situations.

Percentage frequencies were calculated for the closed questions of the two ad hoc questionnaires, while the coding of the open questions involved counting the answers given (e.g., number of memories and emotions reported) and a content analysis, which mainly distinguished between memories with and memories without emotional and relational dimensions.

## 3. Results

### 3.1. Attachment Patterns in Adoptees

The first aim of this study was to determine the distribution of attachment patterns in adoptees. The data showed a clear prevalence of insecure attachment in the sample, with disorganized attachment in particular being the predominant category (N = 16, 40.6%), followed by avoidant (N = 11, 28.1%), ambivalent (N = 6, 15.6%), and secure (N = 6, 15.6%) attachment. We compared our findings with normative data for the adolescent and young adult population derived from a meta-analysis with Italian samples [87] and from an international meta-analysis [88]. As shown in Table 2, the distribution of attachment patterns is significantly different in both cases (*p* < 0.05); the corrected standardized residuals show that D patterns are overrepresented in the sample of adopted young adults, while F patterns are underrepresented. Rather, our findings are consistent with some, but not all, Italian studies on adoptees in which mental representations of attachment were assessed [11,50,89].

Next, we explored the presence of differences based on gender and age at adoption. Regarding the first variable, gender had no effect on the distribution of attachment patterns in either the four-way classification (*Fisher’s Exact Test p* = n.s.) or the two-way classification (secure vs. insecure, *Fisher’s Exact Test* = 2.91; *p* = n.s.), while the effect of age at adoption was demonstrated (two-way classification *Fisher’s Exact Test* = 5.26; df = 2; *p* = 0.04). In accordance with an international meta-analysis of adoptees [32], children with secure attachment were 80% adopted before the age of 6 months and the remaining 20% before the age of one year; on the other hand, the risk of developing an insecure attachment was higher in children who were adopted after the age of one year, i.e., in a period in which the attachment relationship has already been established (Table 3).

Finally, the distribution of attachment patterns was not influenced by the loss of one of the parental caregivers or the divorce of the parents.

### 3.2. Representations of Attachment in Adoptive Parents and Children

The second aim of the study was to determine the mental state of adoptive parents in relation to attachment (Table 4). The secure and dismissing categories were most common in both mothers and fathers. An unresolved mental state was present in around 10% of mothers and just over 7% of fathers. Comparing the results with the main meta-analyses on the non-clinical parental population, a higher percentage of dismissing attachment was observed both in mothers and in fathers. Comparing the data with other studies on Italian couples, the distribution of attachment representations among mothers showed no statistically significant differences; on the contrary, the proportion of dismissing attachments was higher among the fathers in our sample.

The third specific aim of the paper was to analyze the relation between parents’ and children’s mental representations of attachment (Table 5). No statistically significant associations were found: the agreement index between corresponding categories (two categories: secure vs. insecure) did not show significant associations either with respect to mothers (K = 0.069) or with respect to fathers (K = −0.351).

In agreement with Simonelli & Vizzielo [11], we also investigated the relationship between the attachment of the parental couple (both parents secure, only one parent secure, both parents insecure) and the attachment of the children: no statistically significant correlation was found. Finally, we assessed the concordance between maternal and paternal mental representations of attachment; Cohen’s kappa coefficient showed moderate agreement between the mothers’ and fathers’ ratings on the AAI (K = 0.29).

### 3.3. Communication in the Family About Adoption and Children’s Attachment Representation

The final aim of the study was to analyze the presence and quality of communication in the family related to the adoption process and its possible link to the children’s attachment representation. Most children reported that communication with their parents about the adoption experience was absent or poor over the years (N = 29; 73.6%), while only 23.7% (N = 9) reported such communication as frequent and continuous over time. In only 40% of cases did the children state that they were able to talk to both parents.

Parents’ responses, where there were no statistically significant differences between fathers and mothers, also indicated that it was difficult to share the adoption story with their children. In fact, 67.7% of parents who were asked about their memories of the adoption process reported memories of bureaucratic and organizational problems, while only 33.3% reported memories of an emotional and relational nature. In line with this finding, only 39.3% of parents reported that they were able to talk calmly about the adoption with their children, while 32% reported negative emotions such as discomfort and sadness and the remaining 29.6% reported ambivalent emotions.

The analysis revealed an association between children’s attachment patterns and the reappearance of the topic of adoption in family narratives: young adults with an insecure attachment pattern were less likely to tell their parents about memories, emotions, and planning related to the adoption experience (two-way classification system: *χ*^2^ = 12.08; df = 1; *p* < 0.001). There was also evidence of a link between parents’ autobiographical competence in relation to the early stages of relationship building with the adopted child and the children’s attachment. In particular, for children with secure attachment patterns, parents reported memories categorized as felt emotions in 100 percent of cases. For insecure attachment patterns, this percentage dropped to 53.8 percent, with the narratives also referring to concrete and bureaucratic topics (two-way classification system: *χ*^2^ = 15.20; df = 1; *p* < 0.001).

## 4. Discussion

The literature unanimously emphasizes that adoption is a decisive intervention in an often painful and maladjusted development of the child and plays a crucial role in restoring the child’s developmental trajectory. In particular, late-adopted children from international contexts are more likely to have experienced multiple adversities and have been exposed to multiple developmental risk factors that can have a cumulative effect. In these situations, particularly, the opportunity to build new, more secure attachment relationships with the adoptive parents can have a positive impact on developmental progress. Given the importance of the adoption process for the well-being and development of adopted children in both the short and long term, it is important that further research reflects on some aspects that are still controversial. In our study, we therefore sought to examine these aspects in a group of adopted young adults.

The first aim of our work was to assess the distribution of attachment patterns in adopted children, taking into account gender differences and age of adoption. The prevalence of insecure attachments, especially disorganized attachments, differs strongly from normative data referring to the Western population [88,90] and specifically to the Italian population [91,92]. However, the research data on adopted children are controversial. On the one hand, some studies have found that the distribution of attachment patterns among adopted individuals is similar to that of non-adopted and non-vulnerable individuals [47,70,87,93,94]. However, other research highlighted a lower proportion of secure attachments in adopted individuals compared to their peers who grew up in their biological families, and a greater presence of insecure attachments. The results of our study are consistent with this second group of studies. In particular, the high prevalence of insecure attachments is consistent with the findings of Barone and Lionetti [89] and Román et al. [23] on samples of late-adopted children and with the results of the meta-analysis by van den Dries et al. [32] on adoption and foster care context. These authors suggest that the high incidence of disorganization may be related to the adversity experienced prior to adoption. The differences found by age of adoption are consistent with previous research suggesting that adoptees are less likely to form secure attachments after extended periods of deprivation and institutionalization. This effect is particularly pronounced when adoption occurs after the first year, a developmental period during which attachments are formed and when children are particularly vulnerable to caregiving experiences [76,95]: adverse events during the first few months of life, in fact, can have even long-term effects on attachment development. For example, a British longitudinal study [56] compared a group of late-adopted children with a group of children who had been adopted before 12 months and found that late-adopted children showed more indicators of avoidance and disorganization in their narratives, more negative representations of adults and children, and a higher incidence of aggression. The study by Vorria et al. [45] on adopted children who had previously been institutionalized also found higher levels of avoidance behavior. Several studies conducted on the topic of adoption have shown that children with only residential experience have more difficulties after their adoption than children who have had previous family experience [32,96]. As suggested by Steele and colleagues [35], it seems to be easier to incorporate positive representations than to eliminate negative ones. While most improvements in growth and cognitive development occur in the first three years after adoption, recovery in the socioemotional domain may not only take a long time, but may not result in ’earned security‘ unless there is a ’secure‘ adoptive parent in the child’s life who gently helps adopted children let go of their negative thoughts and feelings about their adverse histories.

One possible interpretation for the high percentage of insecure attachment found in our sample, besides the fact that these are individuals who come from institutionalized contexts, can also be linked to the fact that parental attachment style has a distribution that is only partially consistent with studies on attachment in adulthood conducted on both the general population [87] and adoptive families [70,89]. Specifically, in our sample of mothers, although secure and avoidant attachments are most common, as in the normative reference data, the percentage of secure attachments is lower, while that of avoidant attachments is higher. These findings also contradict the claims of Raby et al. [38] that the majority of parents seeking to adopt have developed attachment-related representations that are assumed to promote secure parent–child attachment in the next generation. According to this perspective, adoptive parents’ attachment representations can shape their children’s attachment patterns and support the recovery of attachment quality in children with histories of early adversity. Our results do not confirm the hypothesis of a correlation between the mental representations of attachment in parents and children, contrary to the results of many studies [70,75,97], but they are in line with the results of some other Italian studies [11,94]. Adoptive parents who have insecure attachment states of mind are characterized by difficulties in reconsidering their past emotional experiences with their parents and in processing them into a balanced and coherent representation, as the coding of the AAI shows [86]. This difficulty may be reflected in their inability to help their children organize their autobiographical memories into integrated narratives, as they cannot represent a reliable source of emotional and material support in their children’s upbringing. In our sample, in which the quality of family communication about adoption was analyzed in relation to children’s representations of attachment, it was found that richer and more accurate autobiographical competence of parents in relation to the initial stages of building the relationship with the adopted child tended to be associated with greater security in the children. This confirms that clear and empathic communication about the adoption process, in which experiences and emotions of parents and children are reported, is an important protective factor for adoptees’ well-being and the development of self-representation. Being able to find comfort in what they have experienced allows them to integrate their past into a coherent narrative and can help them to process the early negative experiences they had during the period of institutionalization [70]. The insecure internal working models of late-placed children may therefore be acted out in highly destructive relationship ’scripts‘ in the new family contexts in which they are placed, leading to defiant, passive, hostile, withdrawn and/or rejecting behavior toward the adoptive parents, which can make the experience of parenthood particularly arduous for these couples and require a high level of emotional and reflective competence.

The presence of a high percentage of insecure and disorganized attachments in the young adults of our sample is therefore consistent with some longitudinal studies, in which the percentage of secure attachments increases over time, but only in cases where there is an adoptive parent who provides a secure base that can counteract the effects of institutional care [71,76]. In contrast, when the adoptive parents themselves are fragile on a personal level and in their marital relationship, or are carriers of poorly elaborated elements of their childhood history, or are contending with painful issues from their adult history (e.g., sterility/infertility), highly conflictual and stressful family situations can arise that only reluctantly validate the adoptive children’s insecure internal working models.

It is also conceivable that the presence of non-syntonic attachment models within the parental couple, as found in our study, had an impact on the parenting style according to the spillover hypothesis and created a family emotional environment that was less conducive to the reprocessing of early traumatic events. The spillover hypothesis proposes that negativity aroused in one family sub-system is likely to bleed over into other family sub-systems [98], whereby interparental conflicts would lead to negative parenting and negative parent–child relationship, as numerous studies have consistently highlighted [99]. According to Treboux et al. [100], an optimal synchrony of the parents’ attachment models would have a positive influence on the parenting style.

### Limits

The present study had the advantage of focusing on the long-term outcomes of the adoption process by examining the mental representations of attachment in young adults who were institutionalized early and subsequently adopted by Italian couples. It also had the advantage of analyzing and comparing mental representations of attachment not only in adoptive mothers but also in adoptive fathers. However, the study has some limitations. First, although the number of subjects in our sample is in line with most studies on international adoption, a larger sample would have allowed for a more in-depth statistical analysis. With a larger sample, it would have been possible to test causal hypotheses and examine the moderating or mediating effect of some variables. Although all subjects were institutionalized, their histories may differ in many ways, such as the reason for institutionalization, the age at which they were institutionalized, the quality of the relationship with their biological parents, abuse suffered—all aspects about which we were unable to obtain information. As Howe [71] states, different pre-adoptive experiences lead to the internalization of different attachment representations that identify developmental pathways and behavioral profiles that may differ from one another. For example, it is known that what Howe calls a ‘good start’, i.e., children who enjoyed good quality care from primary caregivers in the first year or two of life before being institutionalized and then adopted, limits the negative impact on the attachment system compared to ‘poor start’ or early institutionalization. In addition, people who were placed at an older age are more likely to report that they did not feel a sense of belonging in their adoptive family when they were growing up and that they did not feel loved by their adoptive parents [76]. It is also known that severe abuse experiences at a young age increase the likelihood of attachment disorders that are difficult to repair by later attachment relationships [1]. Furthermore, there are no data in our study on adoptees’ temperament or cognitive development, variables that may contribute to the difficulties in reworking one’s traumatic history, as difficult temperament or cognitive retardation may be a risk factor for the development of reflective and mentalizing skills [101].

In addition, it would have been useful to have data on the possible presence of psychopathology in the parents and/or the children to better explain the percentages of insecure attachment, which differ not only from the normative data of the general population but also from the results of some longitudinal studies on adoptive families. Indeed, the presence of psychopathological syndromes correlates with the development of insecure attachment relationships, especially disorganized relationships.

Finally, only a longitudinal study with an assessment of IWMs at the time of adoption and later at other developmental milestones would make it possible to test the long-term consequences of early institutionalization and to answer the question of continuity/discontinuity of IWMs. As our study is not longitudinal, the long time that had elapsed since adoption, which should have allowed at least partial change in attachment models in adoptees, is in reality a ’black box‘ of which we know nothing about life events that may have contributed to painting the picture of affective insecurity in our sample.

## 5. Conclusions

Given the fragility of institutionalized and late-adopted children, a high level of parenting quality on the part of the adoptive parents is essential, which should be characterized by sensitivity, responsiveness, reflective function, flexibility, and capacity for change, together with good tolerance of stressful events that may disrupt the couple’s life. With this in mind, it would be important to give adoptive parents the opportunity to participate in parent training that helps them understand the early traumas that adopted children often experience so that they can support their children in their growth and development. Interventions aimed at promoting reflective skills would also help parents feel more confident in talking to their children/young people about their life story and the reasons for their adoption. The most conclusive studies with positive results point to the benefits of early interventions that are preventive rather than reactive and focus on parents’ attunement skills [102]. It is therefore important to design long-lasting parenting support interventions, so that early-deprived children may achieve ’earned security‘. In the absence of a secure base, negative mental representations built up in early childhood are likely to persist for many years after adoption.

## Figures and Tables

**Table 1:** Age of participants at the time of adoption.

	Frequency	Percent
<6 months	15	38.5
6 months–1 year	11	28.2
1–3 years	9	23.1
3–5 years	2	5.1
>5 years	1	2.6
Total	38	97.4
Missing	1	2.6
Total	39	100

**Table 2:** Distribution of IWMs classifications in adoptees: comparison with normative data and studies on adopted adolescents and young adults. Percentages and standardized residuals.

	B/F (%)	A/Ds (%)	C/E (%)	D/U (%)	Fisher’s Exact Test (*p*-Value)
Young adults adopted This study	15.4	28.2	15.4	41.0	
Italian non-clinical adolescents Cassibba et al. [87]	62.2 −5.6	23.8 0.6	9.8 1.1	4.2 8.0	53.83 (*p* < 0.001)
Non-clinical adolescents and young adults van IJzendoorn & Bakermans-Kranenburg [88]	47.3 −3.7	21.0 1	12.1 0.9	19.6 2.9	16.72 (*p* < 0.001)
Adopted adolescents Simonelli & Vizziello [11]	27.5 −1.3	37.5 −0.9	12.5 0.4	22.5 1.8	n.s.
Preschool adopted children Barone, Lionetti [89]	25.0 −0.09	30.0 −0.01	10.0 0.6	35.0 0.4	n.s.
Adopted adolescents Pace et al. [50]	67.4 −4.8	26.1 0.2	6.5 1.3	0 4.8	37.34 (*p* < 0.001)

**Table 3:** Distribution of attachment patterns by age at the time of adoption *.

	Secure Attachment		Insecure Attachment	
Age at the Time of Adoption	N	%	N	%
<6 months	5	83.3	10	31.3
6 months–1 year	1	16.7	10	31.3
>1 years	0	0	12	37.5
Total	6	100	32	100

* The case (N = 1) where the age was unknown was excluded from the analysis.

**Table 4:** Distribution of parents’ mental state in relation to attachment: comparison with normative data and studies on adoptive couples. Percentages and standardized residuals.

		F (%)	Ds (%)	E (%)	U (%)	Fisher’s Exact Test (*p*-Value)
Adoptive parents This study	Mothers	37.9	44.8	6.9	10.3	
	Fathers	51.9	40.7	0	7.4	
Italian non-clinical parents Cassibba et al. [87]	Mothers	59.8 −2.3	18.9 3.3	11.6 −0.8	9.6 0.1	9.62 (*p* = 0.01)
International non-clinical parents van IJzendoorn & Bakermans-Kranenburg [88]	Mothers	55.2 −1.8	16.4 3.9	9.0 −0.4	19.3 −1.2	11.89 (*p* = 0.005)
	Fathers	57.3 −0.5	14.9 3.3	11.2 −1.8	16.6 −1.2	11.73 (*p* < 0.001)
Adoptive parents Simonelli & Vizziello [11]	Mothers	37.5 −1.3	6.3 −0.9	37.5 0.4	18.8 1.8	n.s.
	Fathers	40.0 0.9	26.7 1.1	26.7 −2.9	6.7 0.1	9.2 (*p* = 0.01)
Adoptive parents Barone, Lionetti [89]	Mothers	35.0 0.2	35.0 0.7	20.0 −1.4	10.0 0.0	n.s.
	Fathers	50.0 0.1	35.0 0.4	5.0 −1.2	10.0 −0.3	11.73 (*p* = 0.005)
Adoptive parents Pace et al. [50]	Mothers	70.0 −2.5	6.7 3.4	0 1.5	23.3 −1.3	14.56 (*p* < 0.001)

**Table 5:** Agreement between children’s and parent’s attachment.

	Mother’s Attachment		Father’s Attachment	
Children’s attachment	Secure	Insecure	Secure	Insecure
Secure	8.3%	12.5%	0%	18.2%
Insecure	25.0%	54.2%	45.5%	36.4%

## Data Availability

The data presented in this study are available upon request from the corresponding authors.
